# Changes in Litter Organic Acid Release Characteristics During Litter Decomposition in Plantations Comprising Different Tree Species

**DOI:** 10.3390/plants15071005

**Published:** 2026-03-25

**Authors:** Jiangfei Li, Yanmin Ren, Zhanling Wang, Xiaojian Wu, Kai Wang, Aiqin Liu, Xiangqing Ma

**Affiliations:** 1 College of Forestry, Fujian Agriculture and Forestry University, Fuzhou 350002, China; ljfei9661@126.com (J.L.); 52404022035@fafu.edu.cn (Z.W.); wangkai1072@fafu.edu.cn (K.W.); 2 Chinese Fir Engineering Research Center of National Forestry and Grassland Administration, Fuzhou 350002, China

**Keywords:** plantation forest, low-molecular-weight organic acid, litter decomposition dynamics, forest management, ecological preservation

## Abstract

Phosphorus deficiency restricts the productivity of plantation forests in southern China. Low-molecular-weight organic acids (LMWOAs) can promote insoluble P activation and improve P availability in red soils. However, few studies have investigated organic acids (OAs) released during litter decomposition under field conditions. A one-year litterbag decomposition experiment with monthly sampling was conducted using four common plantation tree species in subtropical China, namely, *Phoebe bournei*, *Michelia macclurei*, *Schima superba*, and *Cunninghamia lanceolata*, to determine changes in LMWOA composition, content, and release characteristics during decomposition. Seventeen LMWOAs were detected during litter decomposition, and OA types differed among tree species. The total amount of litter-derived OAs varied among species, following the order *S. superba* > *P. bournei* > *C. lanceolata* > *M. macclurei*, with the amount in *S. superba* being 1.15 times that in *M. macclurei*. The release characteristics of OAs differed significantly. *C. lanceolata*, *S. superba*, and *M. macclurei* exhibited a net release pattern, whereas *P. bournei* exhibited a release–enrichment–release pattern. *S. superba* and *M. macclurei* litter released significantly more OAs than *C. lanceolata*. Overall, this study provides field-based evidence for interspecific differences in litter-derived OAs dynamics and offers a basis for tree species selection in mixed plantations with potential implications for nutrient return and phosphorus cycling.

## 1. Introduction

The southern forest area of China is dominated by acidic red soil. The aluminum oxide and iron oxide in the soil easily combine with phosphorus to form insoluble phosphates, which are not readily absorbed by plants. This scarcity of available P limits the yield of plantation forests in southern China [1,2,3]. Some plants can activate rhizosphere soil insoluble P and improve soil P bioavailability by increasing the secretion of organic acids (OAs) under P-deficient conditions [4,5]. Therefore, improving the utilization of insoluble P in the southern red soil region in China by optimizing soil OA content has become a major concern in forestry production.

OAs play an important role in nutrient cycling in forest ecosystems [6]. Among them, low-molecular-weight organic acids (LMWOAs) in forest litter mainly include aliphatic acids and aromatic acids with one or more carboxyl groups, such as oxalic acid, citric acid, malonic acid, fumaric acid, benzoic acid, and cinnamic acid. Their relatively simple molecular structures and reactive carboxyl groups enable them to participate actively in nutrient mobilization and phosphorus transformation in soils. Accordingly, previous studies have focused on specific pathways, such as LMWOAs released by roots changing soil conditions and influencing microorganisms to promote the activation of insoluble P in rhizosphere soils [7,8]. Other studies have confirmed that the LMWOAs released during litter decomposition may contribute to soil P release and promote the activation of insoluble P in soil [9,10,11]. However, comparatively less is known about the composition, temporal dynamics, and release patterns of low-molecular-weight organic acids (LMWOAs) during litter decomposition among different plantation tree species under field conditions. This limits our understanding of species-specific litter effects on nutrient return and their potential implications for phosphorus cycling in subtropical plantations.

Chinese fir (*Cunninghamia lanceolata* (Lamb.) Hook.) is a fast-growing timber species widely grown in China. According to the 9th National Forest Inventory of China, the area and volume of Chinese fir forests account for 17.33% and 22.30% of the total area of planted tree forests, respectively, playing a pivotal role in China’s forestry production. However, Chinese fir plantation forests have long suffered from the issue of declining productivity of multi-generation stands, with the insufficiency of soil effective P content playing a major role [12,13,14]. Planting mixed forests with broadleaf species is an effective way to address the declining productivity of continuous Chinese fir plantations [15,16,17]. The positive impact of mixed tree species on soil quality may be due to species complementarities or selection effects [18]. In mixed forests, different tree species interact, leading to ecological differentiation, which can improve litter quality and nutrient balance. Mixed forests also exhibit varying patterns of canopy and root distribution, thereby enhancing the efficiency of utilizing scarce nutrients [19]. Therefore, several scholars have conducted studies on the selection of mixed Chinese fir species from the perspective of ecological niche complementarities, in terms of canopy structure, photosynthesis, and root system, screening a number of broadleaf species that were suitable for establishing mixed plantations with Chinese fir [20,21,22]. However, comparatively few studies have evaluated tree species selection for mixed Chinese fir plantations from the perspective of litter-derived organic acid release and its relevance to phosphorus cycling. Therefore, identifying broadleaf species that differ from Chinese fir in litter organic acid dynamics may provide an ecological basis for species selection in mixed plantations.

To address this gap, we investigated low-molecular-weight organic acid release during litter decomposition in four common plantation tree species in subtropical China, namely, *Phoebe bournei*, *Michelia macclurei*, *Schima superba*, and *Cunninghamia lanceolata*. We hypothesized that organic acid composition and release patterns would vary among species and decomposition stages, and that these differences would be associated with litter nutrient dynamics. This study provides field-based comparative evidence that litter-derived organic acid dynamics differ among common subtropical plantation species and may have distinct implications for nutrient return, phosphorus cycling, and tree-species selection in plantation management.

## 2. Results

### 2.1. LMWOA Species Comparison Among Tree Species

**Figure 1:**
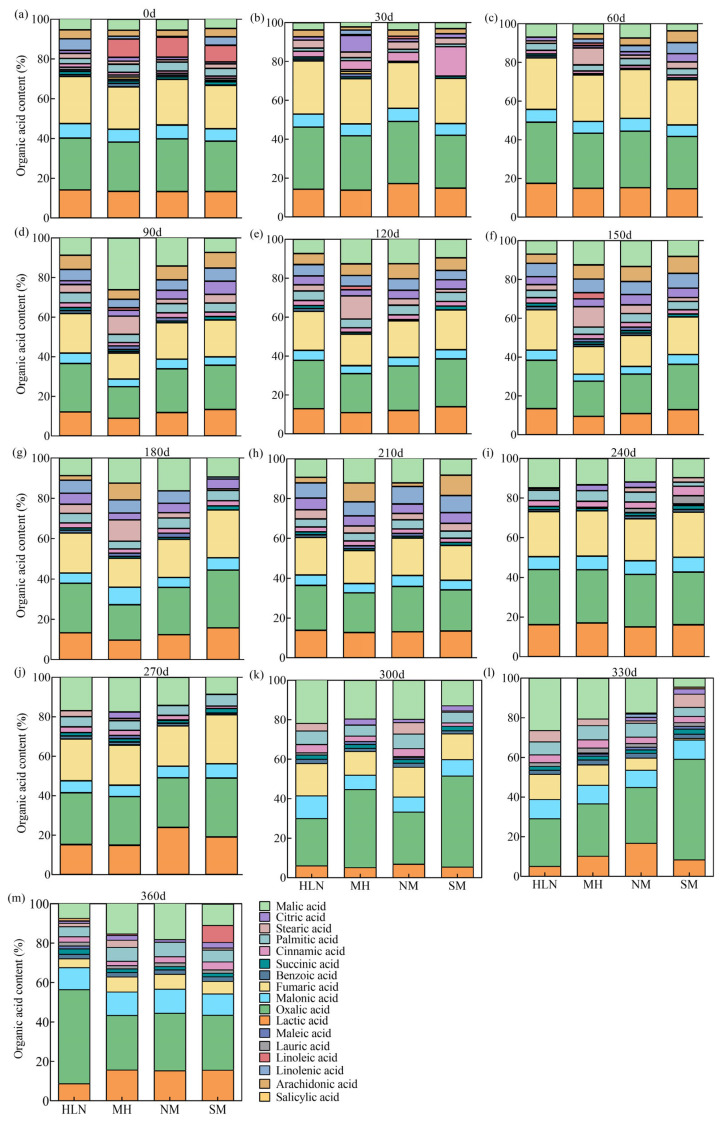
Relative percentage of low-molecular-weight organic acids (LMWOAs) in litter of different tree species. (**a**–**m**) Relative content changes in LMWOAs in the litter of four tree species from 0 to 360 days of decomposition. HLN, *Michelia macclurei*; MH, *Schima superba*; NM, *Phoebe bournei*; SM, *Cunninghamia lanceolata*.

Twenty LMWOAs common in forest litter were analyzed in the litter of different tree species. The results showed that seventeen LMWOAs produced during the decomposition of litter from different tree species were detected, while sorbic acid, tartaric acid and pyruvate were not detected (Figure 1). Nine of these (lactic, oxalic, malonic, fumaric, cinnamic, palmitic, stearic, malic, and succinic acids) were detected in the litter of all four species after 1 year of decomposition. However, the overall LMWOA profile varied greatly among tree species. In addition to the abovementioned nine acids, citric, linolenic, arachidic, maleic, linoleic, and lauric acids were detected in the *C. lanceolata* litter (salicylic and benzoic acids were not present). The OAs detected in *P. bournei* litter were similar to those detected in *C. lanceolata* litter. Citric, linolenic, arachidic, maleic, and benzoic acids, but not salicylic and lauric acids, were detected in the *M. macclurei* litter. All 17 OAs were detected in *S. superba* litter.

### 2.2. Quantitative Characteristics of LMWOAs Released During Litter Decomposition

**Figure 2:**
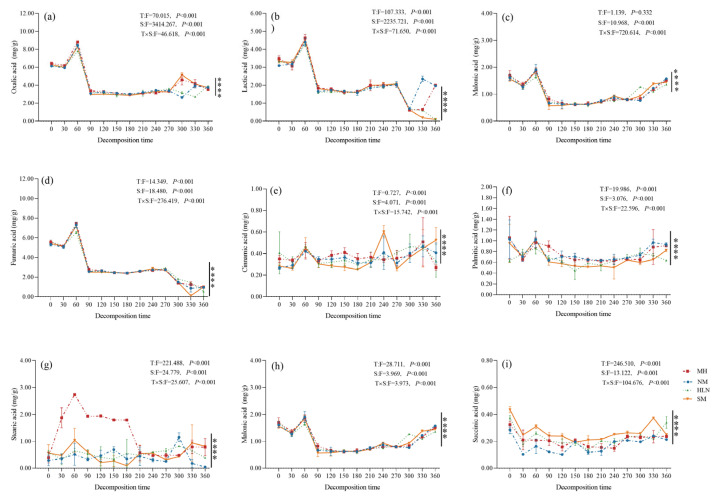
Dynamic changes in the content of oxalic acid (**a**), lactic acid (**b**), malonic acid (**c**), fumaric acid (**d**), cinnamic acid (**e**), palmitic acid (**f**), stearic acid (**g**), malic acid (**h**), and succinic acid (**i**) from the litter of different tree species. HLN, *Michelia macclurei*; MH, *Schima superba*; NM, *Phoebe bournei*; SM, *Cunninghamia lanceolata*. T, Time effect; S, Treatment effect; F, indicates the ratio of mean square between groups to mean square within groups. “****” indicates significant interaction between species.

Figure 2 presents the differences in the quantitative characteristics of the OAs released during litter decomposition. The total amount of OAs in the litter material followed the order *S. superba* > *P. bournei* > *C. lanceolata* > *M. macclurei*, with the total amount in *S. superba* litter being the highest (28.15 mg/g) and 1.15 times that in *M. macclurei* litter. The OA content in the litter of the four tree species was in the following order: oxalic acid > fumaric acid > lactic acid > malic acid > malonic acid > palmitic acid > stearic acid > cinnamic acid > succinic acid. With the progression of decomposition, the total amount of OAs in the litter of all four species showed an initial decreasing trend, followed by an increasing and then decreasing trend. The total amount of OAs in the pre-decomposition period (days 0–90) across the four species showed the following order: *S. superba* > *C. lanceolata* > *M. macclurei* > *P. bournei*. In the mid-decomposition period (days 120–270), we observed the following order: *S. superba* > *P. bournei* > *M. macclurei* > *C. lanceolata*. In the late decomposition period (days 300–360), we observed the following order: *C. lanceolata* > *P. bournei* > *S. superba* > *M. macclurei*.

We observed significant differences in the amounts of each type of OA detected in the litter materials of the four species. The succinic acid content was the highest in the initial stage of decomposition of *C. lanceolata* litter, which was significantly higher than that observed in other decomposition periods and other species. The malic acid content was highest at day 30; lactic, oxalic, fumaric, malonic, and palmitic contents were highest at day 60; cinnamic acid content was highest at day 240; and stearic acid content was highest at day 330. Succinic acid content in the litter of *P. bournei* was highest in the initial stage of decomposition, showing the same pattern as that noted for *C. lanceolata.* Oxalic, lactic, malonic, fumaric, stearic, and palmitic acid contents were highest at day 60. Malic and cinnamic acid contents were highest at day 330. Succinic acid contents were highest in *M. macclurei* litter in the initial stage of decomposition. Oxalic, lactic, malonic, fumaric, cinnamic, and palmitic acid contents were highest at day 60. Stearic acid content was highest at day 300; at day 330, malic acid content was significantly higher than that observed during other decomposition periods. In the case of *S. superba*, succinic and palmitic acid contents were higher in the initial stage of decomposition; oxalic, lactic, malonic, fumaric, and stearic acid contents were highest at day 60. Furthermore, malic acid content was highest at day 90, and cinnamic acid content was highest at day 330.

### 2.3. Release Characteristics of LMWOAs During Litter Decomposition

**Figure 3:**
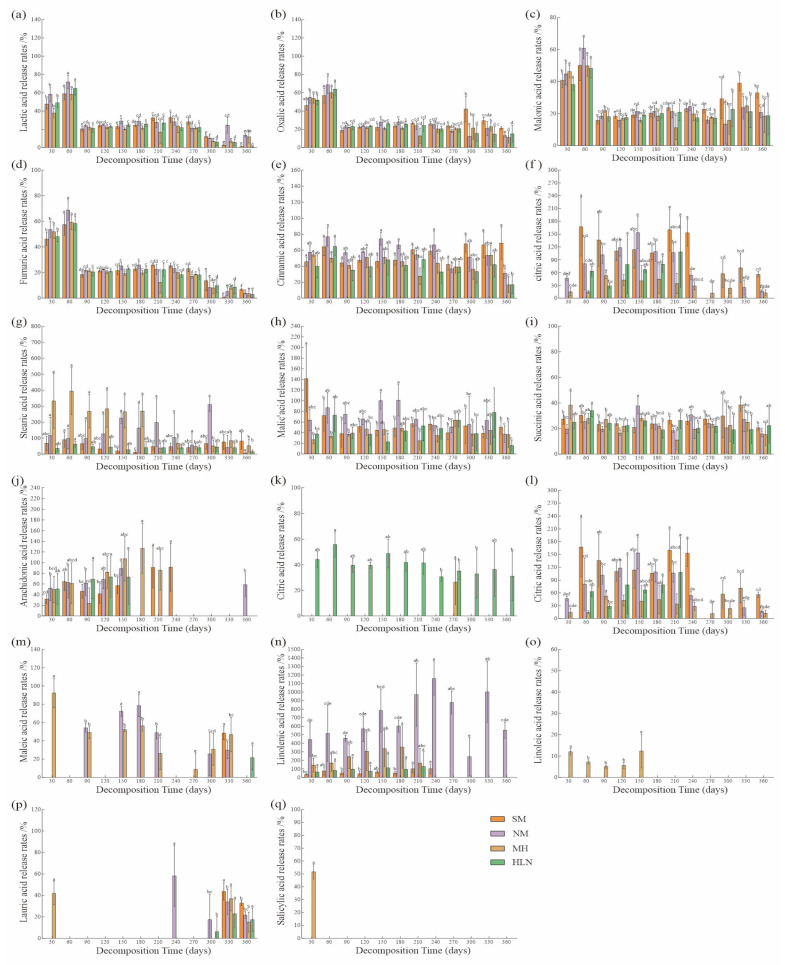
Release characteristics of oxalic acid (**a**), lactic acid (**b**), malonic acid (**c**), fumaric acid (**d**), cinnamic acid (**e**), palmitic acid (**f**), stearic acid (**g**), malic acid (**h**), succinic acid (**i**), citric acid (**j**), linolenic acid (**k**), arachidonic acid (**l**), maleic acid (**m**), linoleic acid (**n**), benzoic acid (**o**), lauric acid (**p**), and salicylic acid (**q**) from the litter samples of four tree species. HLN, *Michelia macclurei*; MH, *Schima superba*; NM, *Phoebe bournei*; SM, *Cunninghamia lanceolata*. Different lowercase letters indicate significant (*p* < 0.05) differences in the low-molecular-weight organic acid content release rates of the litter samples of the same tree species at different decomposition periods.

The release characteristics of LMWOAs during litter decomposition significantly differed among species (*p* < 0.05; Figure 3). During *C. lanceolata* decomposition, oxalic, malonic, fumaric, palmitic, malic, succinic, arachidic, maleic, and lauric acids were in a net release state (release rate, 12.89–98.99%), whereas lactic, cinnamic, stearic, citric, and linolenic acids were in an enriched state. During *P. bournei* decomposition, oxalic, lactic, malonic, fumaric, palmitic, cinnamic, succinic, arachidic, maleic, and lauric acids were in a net release state (release rate, 3.84–95.71%), whereas stearic, malic, citric, and linolenic acids were in an enriched state. During *S. superba* decomposition, oxalic, lactic, malonic, fumaric, palmitic, cinnamic, malic, citric, succinic, and linoleic acids were in a net release state (release rate, 32.54–94.28%), whereas stearic and linolenic acids were in an enriched state. During *M. macclurei* decomposition, oxalic, lactic, malonic, fumaric, cinnamic, palmitic, malic, succinic, and benzoic acids were in a net release state (release rate, 24.35–92.36%), whereas linolenic acid was in an enriched state; citric acid showed an initial trend of net release state, followed by an enrichment state.

The release patterns of the LMWOAs at different stages of decomposition were significantly different (*p* < 0.05; Figure 3). Citric acid was in an enriched state during days 0–180 in *C. lanceolata* litter, with a high enrichment rate (88.05%). Cinnamic and lactic acids were in an enriched state at days 240 and 300, respectively, with enrichment rates of 41.13% and 10.99%, respectively. In *P. bournei* litter, all OAs were in an enrichment state; the enrichment rate of citric acid reached 68.62% after day 150; malic acid was enriched by 10.88% after day 180; linolenic acid was enriched by the highest rate (1171.97%) after day 240; and stearic acid was enriched by the highest rate (280.65%) after day 300. In *S. superba* litter, stearic, arachidic, and linolenic acids were enriched at 30–210 days. The enrichment rate of stearic acid reached 344.22% at day 60, whereas that of linolenic and arachidic acids was highest at day 180 (298.27% and 41.48%, respectively). In *M. macclurei* litter, at day 210, the enrichment rates of citric and linolenic acids were 21.72% and 45.08%, respectively; at day 330, the enrichment rate of malic acid was 7.51%. The remaining OAs exhibited a net release state at days 30–360.

### 2.4. Nutrient Characteristics During Litter Decomposition

**Figure 4:**
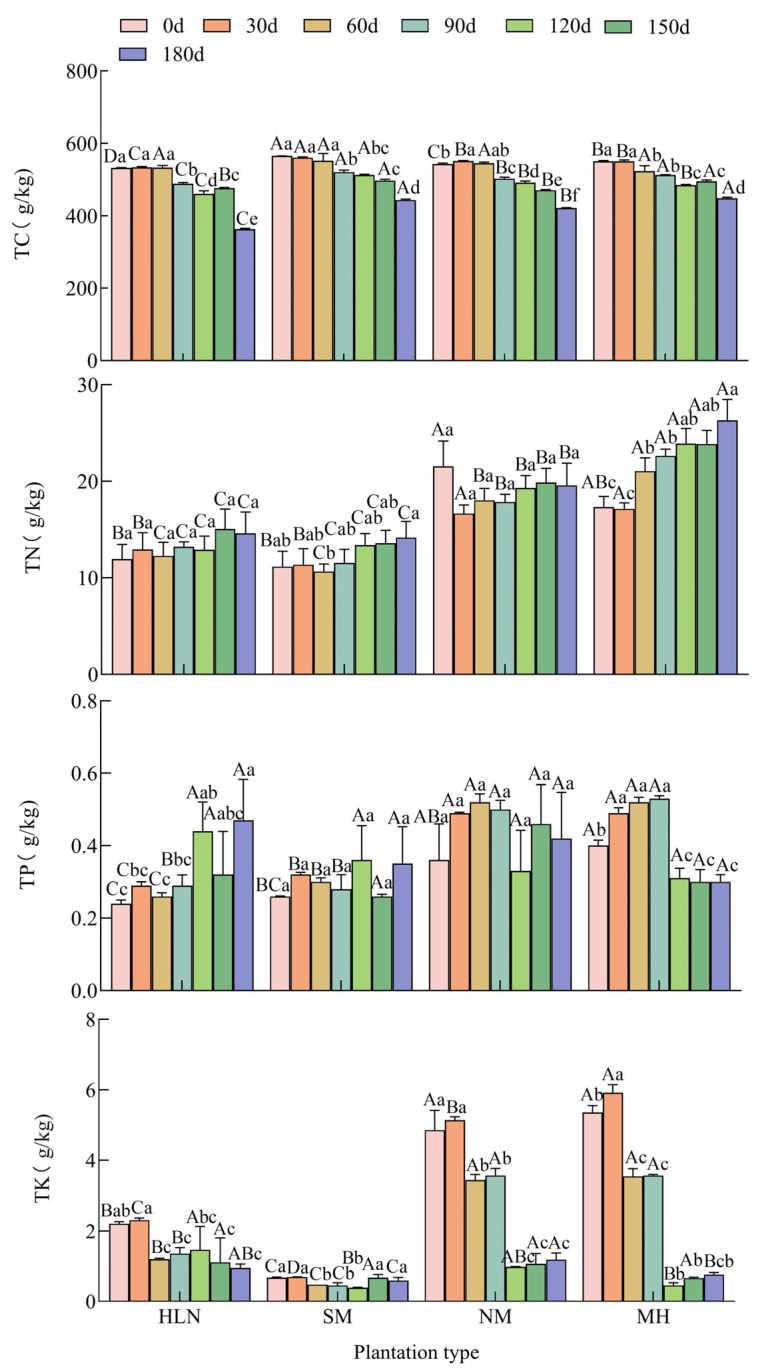
Changes in the nutrient content of litter during decomposition. TC, total carbon; TN, total nitrogen; TK, total potassium; TP, total phosphorus; HLN, *Michelia macclurei*; MH, *Schima superba*; NM, *Phoebe bournei*; SM, *Cunninghamia lanceolata*. Different lowercase letters indicate significant differences in the nutrient content of the litter samples of the same tree species at different decomposition periods, and different uppercase letters indicate significant differences in the nutrient content of litter from different tree species during the same decomposition period (*p* < 0.05).

Based on the monthly dynamics of litter nutrient characteristics during decomposition (Figure 4), significant interspecific differences were observed in C, N, P, and K contents (all *p* < 0.05). Overall, C content followed the order *C. lanceolata* > *S. superba* > *P. bournei* > *M. macclurei*, and showed a general declining trend during decomposition, with *C. lanceolata* maintaining relatively high values during the early and middle stages. In contrast, N content followed the order *S. superba* > *P. bournei* > *M. macclurei* > *C. lanceolata*. The N content generally increased over time in *C. lanceolata*, *S. superba*, and *M. macclurei*, whereas *P. bournei* showed an initial decrease followed by an increase.

Significant interspecific differences were also found in P and K contents (both P < 0.05). Overall, P content followed the order *P. bournei* > *S. superba* > *M. macclurei* > *C. lanceolata*, with *P. bournei* and *S. superba* showing relatively higher values during the early decomposition stage, whereas *M. macclurei* became higher at some middle and late stages. K content followed the order *S. superba* > *P. bournei* > *M. macclurei* > *C. lanceolata*. Higher K contents were observed in *P. bournei* and *S. superba* during the early stage, while *M. macclurei* showed relatively higher K content during the middle stage. These results indicate clear interspecific differences in nutrient dynamics during litter decomposition.

### 2.5. Correlation Analysis Between LMWOAs and Nutrient Contents

**Figure 5:**
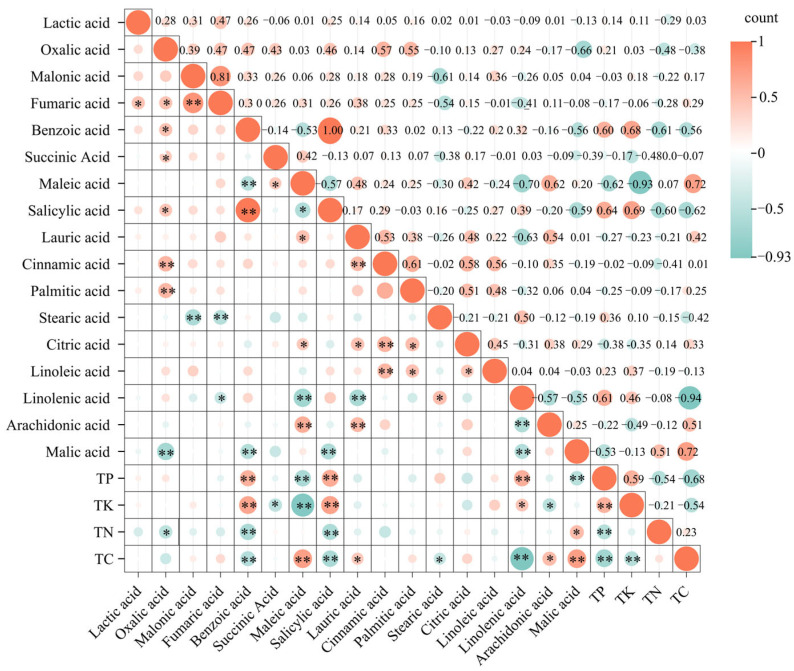
Correlation between low-molecular-weight organic acids and nutrient contents during litter decomposition. TC, total carbon; TN, total nitrogen; TK, total potassium; TP, total phosphorus. * and ** indicate significant correlation (*p* < 0.05) and extremely significant correlation (*p* < 0.01), respectively.

We observed a significant correlation between the LMWOAs and nutrient contents during the decomposition process (Figure 5). The contents of total P and benzoic, salicylic, and linoleic acids were significantly and positively correlated (*p* < 0.01), and a highly significant negative correlation was noted with the maleic and arachidic acid contents (*p* < 0.01). TK content showed a highly significant positive correlation with benzoic and salicylic acids (*p* < 0.01), highly significant negative correlation with maleic acid (*p* < 0.01), highly significant positive correlation with linoleic acid (*p* < 0.05), and highly significant negative correlation with succinic and linolenic acids (*p* < 0.05). TN content showed a highly significant negative correlation with benzoic and salicylic acids (*p* < 0.01), significant positive correlation with arachidic acid (*p* < 0.05), and significant negative correlation with malic acid (*p* < 0.05). TC content showed a highly significant positive correlation with maleic and arachidic acids (*p* < 0.01), highly significant negative correlation with benzoic, salicylic, and linoleic acids (*p* < 0.01), highly significant positive correlation with lauric and linolenic acids (*p* < 0.05), and highly significant negative correlation with palmitic acid (*p* < 0.05).

We noted significant correlations between different nutrients across the litter materials of the four tree species, with a highly significant positive correlation noted between TP and TK (*p* < 0.01) and a highly significant negative correlation between TN and TC (*p* < 0.05). TP was significantly and negatively correlated with TC (*p* < 0.05).

## 3. Discussion

### 3.1. Differences in OAs of Leaf Litter from Different Tree Species

The OAs released during litter decomposition influence nutrient cycling and soil nutrient availability in forest ecosystems [23]. In previous studies on the litter material of *Pinus ponderosa* [24], Dipterocarpaceae [25], and *Abies fabri* [26], different types of OAs were observed. The types of OAs secreted by the roots of different tree species vary, with broadleaf deciduous trees, broadleaf evergreens, and shrubs mainly secreting citric, malic, oxalic, and succinic acids, and conifers mainly secreting oxalic acid [27]; coniferous species mainly secrete citric acid, along with malic, tartaric, and oxalic acids [26], whereas broadleaf species such as poplar mainly secrete palmitic and lauric acids [28]. In this study, we identified 17 OAs released during the decomposition of *C. lanceolata*, *P. bournei*, *M. macclurei*, and *S. superba* litter. Oxalic acid content was the highest across the four species, followed by fumaric and lactic acids; oxalic acid content in *C. lanceolata* litter was significantly higher than that in the other species, which agrees with the results of Chen et al. [27], who concluded that conifers mainly secrete oxalic acid. However, in the *C. lanceolata* samples, the total amount of OAs was significantly lower than that in the *S. superba* and *P. bournei* samples, similar to the results of Zou [11], who reported that the content of litter OAs in *C. lanceolata* forests was lower than that in broadleaf species (such as water hyacinth and birch). We noted significant differences in the litter OA types and contents across the four tree species, which may be attributed to plant species, natural environment, and cultivation measures [29,30]. Previous research from our group has revealed variations in the composition and content of soil organic acids in plantations of *C. lanceolata*, *P. bournei*, *M. macclurei*, and *S. superba*. These differences may be attributed to variations in the types, concentrations and metabolic activities of the organic acids released from the litter of these tree species into the soil [31]. In this study, the increase in foreign matter (such as animals and roots) in the litter decomposition bags as decomposition progressed affected not only the rate of litter decomposition but also the composition and content of LMWOAs.

The OA content in litter can reduce soil pH and promote the release of fixed P (e.g., Fe-P, Al-P, and Ca-P) by chelating metal ions such as iron and aluminum [32]. Oxalic acid may interact with citric and malic acids to form a multi-coordination system that could significantly enhance the activation efficiency of soil P [33]. Although plants and microorganisms can secrete a variety of LMWOAs, citric acid can extract more P than other OAs and can better promote the release of available P [34]. In our study, citric acid was consistently detected as an important component of the LMWOAs released during litter decomposition, suggesting that it may have contributed to the observed changes in P availability. This is in line with the results of Deng et al. [32], who reported that introducing broad-leaved tree species with higher litter quality and underground secretions such as citric acid into coniferous plantations significantly increased soluble P content in monoculture forest soils. In the present study, malic acid was not detected in the middle stage of decomposition, and citric acid was not detected in the late stage of decomposition of *C. lanceolata* litter. However, throughout the decomposition process, both acetic and lactic acid were detected in *S. superba*. The hybridization of coniferous and broadleaf species could lead to the production of mixed litter with higher decomposition rates and faster release of different OAs [32,35]. Ren et al. [31] found that the total concentration of OAs in the soils of *P. bournei* and *S. superba* plantations was significantly higher than that in *C. lanceolata* plantations. This difference may be attributed to the greater cumulative biomass and litter decomposition rates of the broadleaf species and their understory vegetation compared to *C. lanceolata*.

In the present study, the OA content in *S. superba* litter was significantly higher than that in *C. lanceolata*, *P. bournei*, and *M. macclurei* litter. The OA content in *S. superba* litter was higher during the pre-decomposition period, whereas that in *C. lanceolata* litter was higher during the post-decomposition period. This implies that the OA content of *C. lanceolata* complements that of *S. superba*. Therefore, the selection of mixed plantations between *S. superba* and *C. lanceolata* with non-overlapping ecological niches could increase the type and amount of OAs in the litter materials of different tree species in mixed forests. This may be beneficial for the activation of insoluble P in the soil, thereby facilitating the improvement of *C. lanceolata* plantation productivity.

### 3.2. Characteristics of OA Release from Dried Leaves of Different Tree Species

The decomposition process of forest litter material includes leaching, enriched release, and direct release of the constituents. Different types of OAs are released from the litter material of different tree species during decomposition. These OAs enter the soil through leaching, thereby affecting soil nutrient availability [36,37]. In the present study, the OAs released from the litter of *C. lanceolata*, *S. superba*, and *M. macclurei* showed a net release pattern, whereas those from *P. bournei* showed a release–enrichment–release pattern. The release rates of OAs from *S. superba* and *M. macclurei* were significantly higher than those from *C. lanceolata*, which may be related to the relatively high tannin content and slower decomposition characteristics of *C. lanceolata* litter. Previous studies have shown that *C. lanceolata* litter decomposes more slowly than broad-leaved species such as *P. bournei* and *S. superba*, which may in turn delay the release of organic acids [38].

The decomposition rate of litter in mixed forests that include *C. lanceolata* is significantly higher than that observed in pure forests [39,40], which may be attributed to the fact that the richness of microbial flora in the litter of mixed forests is higher than that in pure forests. Mixed litter can increase microbial diversity by providing diverse ecological niches for microbial decomposers. However, this largely depends on the composition of the tree species present in the mixed forest [41]. Kou et al. [42] demonstrated that the decomposition rate of litter from multiple tree species was faster, and the release rate of OAs in the litter also increased. Bai et al. [43] confirmed that mixed-type litter has a higher rate of litter decomposition and a higher pH. In the present study, the enrichment and release of OAs were dynamic during litter decomposition. OA release was faster in the early stage, fluctuated to different degrees in the middle stage, and then accelerated in the late stage, similar to the results of Zhang [44]. Zhang et al. [5] also reported that OA levels increased significantly during the early stage of forest litter decomposition. These results suggest that OAs may participate in nutrient transformation at different decomposition stages and may influence the timing of nutrient release. In the pre-decomposition period, the release rate of OAs from the litter of different tree species showed the following pattern: *M. macclurei* > *C. lanceolata* > *S. superba* > *P. bournei*. In the post-decomposition period, the release rate of OAs from the litter of *C. lanceolata* was significantly greater than that from the other species. Furthermore, this study found that nutrient release from *C. lanceolata* litter was lower than that from other broad-leaved tree species. A close relationship was observed between low-molecular-weight organic acids and endogenous nutrients in the litter of broad-leaved species. This result suggests that differences in OA dynamics among tree species may affect species-specific nutrient return patterns and further influence phosphorus mobilization and nutrient cycling in plantation ecosystems. Specifically, total phosphorus content was highly significantly positively correlated with benzoic acid, salicylic acid, and linoleic acid; total potassium content was highly significantly positively correlated with benzoic acid and salicylic acid; and total nitrogen content showed a significant positive correlation with arachidic acid. These patterns may reflect the important metabolic functions of low-molecular-weight organic acids in plants [45]. Therefore, the selection of *S. superba*, *M. macclurei*, and *C. lanceolata* for mixed planting could improve litter composition and promote the release of different types of OAs, which could enhance soil nutrient availability in mixed forests.

It should also be noted that litter decomposition in this study was evaluated using 40-mesh litterbags, which may have limited the access of larger soil fauna and thus may not fully reflect natural decomposition processes under field conditions. Therefore, the observed patterns of organic acid composition and release should be interpreted primarily as the result of decomposition processes mediated by microorganisms and smaller decomposers. Nevertheless, because the same litterbag method was used consistently across all four tree species, the interspecific differences observed in this study remain meaningful for comparative analysis.

This study investigated the composition and release characteristics of OAs in the decomposed leaves of different tree species, as well as their relationships with nutrients. The findings provide a theoretical framework for the activation of soil P driven by fallen leaves. In the future, it is necessary to combine metagenomics and stable isotope techniques to reveal the microbial-OA-P coupling mechanism and promote the precision of ecological management in artificial forests.

## 4. Materials and Methods

### 4.1. Study Area

The experimental site was in the Xin Kou Teaching Forest Farm of the Fujian Agriculture and Forestry University in Sanming City, Fujian Province, China (117°54′97″ E, 26°16′79″ N). The region has a subtropical monsoon climate with abundant light, heat, and rainfall, with an average annual temperature being 19 °C and the average annual precipitation being 1749 mm. The topography is characterized by low mountains and hills, and the soil type is mountainous red soil developed from phyllite. Details of the planted forests of *C. lanceolata*, *P. bournei*, *M. macclurei*, and *S. superba* selected in this study are shown in Table 1. Based on our research group’s preliminary analysis of soils from these plots, total phosphorus content and soil organic acid composition differed among plantations. Eight organic acids, namely, oxalic acid, lactic acid, fumaric acid, malonic acid, citric acid, palmitic acid, stearic acid, and cinnamic acid, were detected, and their total concentrations were ranked as follows: *P. bournei* > *S. superba* > *C. lanceolata* > *M. macclurei* [31].

### 4.2. Sample Collection

In December 2020, three independent standard plots (20 m × 20 m) were established for each plantation species, namely *Cunninghamia lanceolata*, *Phoebe bournei*, *Michelia macclurei*, and *Schima superba*. Within each plot, three litter collection frames (1 m × 1 m) were randomly placed to collect freshly fallen leaves. Litter collected from the three frames within the same plot was pooled to obtain one composite sample per plot. Thus, three plot-level biological replicates were obtained for each species. All samples were transported to the laboratory and air-dried under natural ventilation in the shade before use in the decomposition experiment.

### 4.3. Decomposition Test

In December 2020, after air-drying and cleaning, 30 g of litter from each species was weighed and placed into a nylon mesh litterbag (40-mesh aperture, 45 cm × 45 cm). A total of 72 litterbags were prepared for each species, resulting in 288 litterbags in total.

In early January 2021, three 3 m × 3 m decomposition subplots were established within each plantation, corresponding to the three replicate plots. Before litterbag placement, surface debris was carefully removed from each subplot. The litterbags were then placed on the forest floor in the corresponding plantation, laid flat in close contact with the soil surface, fixed at the corners, and marked for subsequent retrieval. Within each subplot, litterbags were evenly distributed to avoid overlap and excessive crowding. To reduce the influence of microsite heterogeneity, litterbags were distributed across the three independent subplots within each plantation rather than concentrated in a single location. The litter was allowed to decompose under natural field conditions for one year.

For statistical analysis, each plot was treated as one biological replicate, and the three plots for each species were used as independent replicates.

### 4.4. Measurement Methods

From January 2021 to January 2022, the decomposition bags were collected at the end of every month. Six bags of litter were collected for each tree species each month. The retrieved bags were sorted to separate the soil and mud and remove fine roots, worms, worm eggs, and sediment. The remaining samples were weighed after shade-drying in a ventilated place. Then, 4–5 g of decomposed litter materials was removed, crushed, and stored in the refrigerator at −20 °C for analyzing the low-molecular-weight OA (LMWOA) content in the leaves. The remaining material was oven-dried at 65 °C to a constant weight to determine the contents of total phosphorus (TP), total nitrogen (TN), total carbon (TC) and total potassium (TK) content.

#### 4.4.1. Litter OA Content Determination

Samples were prepared for determining litter OA content following previous studies [11,31]. Methanol immersion and esterification were performed. Then, 1 g of sample was placed into a 50-mL reaction bottle, and 20 mL of a sulfuric acid–methanol solution (volume ratio of 7:100) was added; the mouth of the bottle was closed with a stopper, and the bottle was shaken. Then, the reaction bottle was placed in a water bath at a constant temperature (65 °C) for 24 h for methyl esterification. Then, 40 mL of pure water was added before shaking and filtering. The filtrate was poured into a 250 mL pear-shaped separating funnel, and 10 mL of chloroform was added before tilting the funnel at 45° and shaking it. After the sample was stratified, the lower layer was first extracted and then dried using a glass column containing anhydrous sodium sulfate. The extracted samples were filtered through a 0.22-μm needle filter into a 2-mL vial for chromatographic analysis.

Preparation of 100 mg/mL individual standard stock solutions: weigh 250 mg of each organic acid standard substance and transfer it into a 25 mL volumetric flask. Then dissolve them with anhydrous methanol and mix well. Fifteen individual organic acid standard stock solutions with a concentration of 100 mg/mL are obtained in total. Pipette 3.33 mL of each of the 15 individual standard stock solutions sequentially into 50 mL flask. Dilute to the 50 mL mark with anhydrous methanol and mix thoroughly. Serial dilutions were prepared from stock solution to obtain concentrations of 0.2, 1, 5, 20, and 50 mg/mL for standard curve.

An Agilent-7890N gas chromatograph with a flame ionization detector (Agilent Company, Santa Clara, CA, USA) was used for measuring LMWOAs in litter. According to the results of previous studies, 20 LMWOAs common to forest soil were selected as standard samples: lactic, oxalic, sorbic, malonic, fumaric, benzoic, succinic, maleic, salicylic, lauric, cinnamic, palmitic, stearic, citric, linoleic, arachidonic, tartaric, pyruvic, malic, and linolenic acids [46].

For the gas chromatograph experiments, a chromatographic column (30 m × 0.25 mm × 0.25 µm; Agilent DB-FastFAME; Agilent Company) was applied with a sample size of 1 μL. The shunt ratio was 30:1 and the inlet temperature was 250 °C. Nitrogen (purity ≥ 99.999%) was used as the carrier gas. The column flow rate was set at 1 mL/min, under constant-flow conditions. The initial temperature was 50 °C (maintained for 2 min), increased to 100 °C (3 °C/min; maintained for 0 min) and 220 °C (20 °C/min; maintained for 5 min). The detector temperature was set to 260 °C, and the flow rates of hydrogen, air, and make-up gas (N2) were set at 30, 400, and 25 mL/min, respectively.

The reagents and drugs used in the test were provided by Fuzhou Miley Biotechnology Co., Ltd., Fujian, China, and were of superior purity. The water used in the experiments was purified. The recovery of the OAs from the litter samples was 93.14–102.17% (based on the spiking method), which met the experimental requirements.

#### 4.4.2. Identification and Measurement of Nutrients in Litter

The nutrients in the litter were identified following Lu [47]. The total P (TP) and total K (TK) were determined using inductively coupled plasma mass spectrometry (PerkinElmer, Richmond, CA, USA) after nitric acid–hydrogen peroxide digestion. The total C (TC) and total N (TN) were determined using a C and N analyzer (Elemantar vario MAX CN, Elementar, Germany).

### 4.5. Data Processing and Statistical Analysis

The release rate of the OAs from litter was calculated as follows [48]:

L = (Mt − 1Xt − 1 − MtXt)/(M0X0) × 100%

where M0 denotes the initial litter dry mass (g), Mt is the residual litter dry mass after decomposition on day t (g), X0 is the OA content in the initial samples (mg/g), and Xt is the OA content of the litters sampled at time t (mg/g). L was used to determine the leaching, release, and consumption of OAs from the litter materials during the litter de-composition process. Negative or near-zero values of L indicate that OAs are decomposed or immobilized, showing a net enrichment of OAs. Positive values of L indicate that OAs release exceeds retention, indicating a net release of OAs from litter.

Microsoft Excel 2010 was used for statistical analysis and graphing. One-way analysis of variance and multiple comparisons were performed using the Statistical Package for the Social Sciences (SPSS 23.0) software. Before data analysis, the normality and homogeneity of variance were tested, and data that did not meet these assumptions were log-transformed to satisfy the requirements of statistical testing. All data are expressed as mean ± standard error. The online ChiPlot tool (https://www.chiplot.online/, accessed on 1 January 2026.) and Spearman correlation analysis (correlation heatmaps) were used to analyze the correlation between various nutrient parameters and the LMWOAs in the samples.

## 5. Conclusions

Our study revealed clear differences in OA content and release characteristics during litter decomposition of *P. bournei*, *M. macclurei*, *S. superba*, and *C. lanceolata*. In these species, 17 LMWOAs were detected, and the total amount followed the order: *S. superba* > *P. bournei* > *C. lanceolata* > *M. macclurei*. The OAs released from the litter of *C. lanceolata*, *S. superba*, and *M. macclurei* showed a net release trend during the decomposition process, whereas those from the litter of *P. bournei* showed a release–enrichment–release trend. The release rate of OAs from the litter of *S. superba* and *M. macclurei* was significantly higher than that of *C. lanceolata*. These interspecific differences in OA dynamics may influence nutrient return and the biological availability of soil P. Overall, our results indicate that species-specific litter plays an important role in regulating soil phosphorus availability, and that optimizing litter composition may help enhance soil fertility and phosphorus cycling. These findings provide a theoretical basis for efficient soil P utilization and for developing ecological restoration and nutrient management strategies in subtropical plantations and other red-soil regions. Among the tested species, mixing *C. lanceolata* with broad-leaved species, especially *S. superba*, may be a promising option for mixed plantations, given the relatively high OA content and net release pattern observed in *S. superba* litter. Such a combination may enhance complementarity in litter-derived OA dynamics and potentially promote phosphorus cycling and nutrient return in subtropical red-soil plantations.

Although this study contributes to understanding the release characteristics of OAs in subtropical plantations, further studies are needed in different geographical regions to comprehensively understand the changing trends of OAs. Future studies should also combine metagenomics with stable isotope techniques to reveal the interaction mechanisms between microorganisms and OAs and their impact on the recycling of forest leaf nutrients. This will deepen our understanding of the efficient P cycling mechanism in subtropical plantation soils.

## Figures and Tables

**Table 1:** Plantation stands of the four different tree species considered in this study.

Tree Species	Year (a)	DBH (cm)	TH (cm)	Stand Density (Plant/hm^2^)	Slope (°)	Elevation (m)	Canopy Density	Longitude (E)	Latitude (N)
*Cunninghamia lanceolata*	42	23.55	21.24	1300	18	324	0.8	117°47′57″	26°16′01″
*Phoebe bournei*	56	24.54	21.39	800	16	211	0.85	117°46′69″	26°16′70″
*Michelia macclurei*	42	34.29	16.50	400	18	276	0.85	117°47′53″	26°15′85″
*Schima superba*	54	25.51	21.40	800	17	219	0.7	117°46′52″	26°16′67″

Note: DBH, diameter at breast height; TH, tree height.

## Data Availability

The original contributions presented in the study are included in the article material; further inquiries can be directed to the corresponding author.

## Abbreviations

DBH	Diameter at breast height
TH	Tree height
LMWOAs	Low-molecular-weight organic acids
OAs	Organic acids
TP	Total phosphorus
TN	Total nitrogen
TC	Total carbon
TK	Total potassium
